# Admission Dehydration Status Portends Adverse Short-Term Mortality in Patients with Spontaneous Intracerebral Hemorrhage

**DOI:** 10.3390/jcm10245939

**Published:** 2021-12-17

**Authors:** Felix Lehmann, Lorena M. Schenk, Joshua D. Bernstock, Christian Bode, Valeri Borger, Florian Gessler, Erdem Güresir, Motaz Hamed, Anna-Laura Potthoff, Christian Putensen, Matthias Schneider, Julian Zimmermann, Hartmut Vatter, Patrick Schuss, Alexis Hadjiathanasiou

**Affiliations:** 1 Department of Anesthesiology and Intensive Care, University Hospital Bonn, 53127 Bonn, Germany; christian.bode@ukbonn.de (C.B.); Christian.Putensen@ukbonn.de (C.P.); 2 Department of Neurosurgery, University Hospital Bonn, 53127 Bonn, Germany; Lorena_Maria.Schenk@ukbonn.de (L.M.S.); Valeri.Borger@ukbonn.de (V.B.); erdem.gueresir@ukbonn.de (E.G.); motaz.hamed@ukbonn.de (M.H.); anna-laura.potthoff@ukbonn.de (A.-L.P.); matthias.schneider@ukbonn.de (M.S.); hartmut.vatter@ukbonn.de (H.V.); patrick.schuss@ukb.de (P.S.); alexis.hadjiathanasiou@ukbonn.de (A.H.); 3 Department of Neurosurgery, Brigham and Women’s Hospital, Harvard Medical School, Boston, MA 02115, USA; jbernstock@partners.org; 4 Department of Neurosurgery, University Hospital Rostock, 18055 Rostock, Germany; Florian.Gessler@med.uni-rostock.de; 5 Department of Neurology, University Hospital Bonn, 53127 Bonn, Germany; julian.zimmermann@ukbonn.de

**Keywords:** spontaneous intracerebral hemorrhage, dehydration, mortality, fluid balance

## Abstract

The impact of dehydration at admission of patients with spontaneous intracerebral hemorrhage (ICH) on short-term mortality remains ambiguous due to scarce data. All of the consecutive patients with spontaneous ICH, who were referred to our neurovascular center in 2018/19, were assessed for hydration status on admission. Dehydration was defined by a blood urea-to-creatinine ratio > 80. In a cohort of 249 patients, 76 patients (31%) were dehydrated at the time of admission. The following factors were significantly and independently associated with increased 30-day mortality in multivariate analysis: “signs of cerebral herniation” (*p* = 0.008), “initial midline shift > 5 mm” (*p* < 0.001), “ICH score > 3” (*p* = 0.007), and “admission dehydration status” (*p* = 0.007). The results of the present study suggest that an admission dehydration status might constitute a significant and independent predictor of short-term mortality in patients with spontaneous ICH.

## 1. Introduction

An intracerebral hemorrhage (ICH) is one of the most fatal forms of stroke. Depending on the localization (deep-seated versus lobar), different causes (e.g., hypertension, arteriovenous malformations, aneurysms, tumors) may account for such hemorrhage [1,2]. Despite a variety of potential therapeutic approaches (e.g., blood pressure control, aspiration, decompressive craniectomy), the outcome (in this case, 30-day mortality) of ICH is often considered poor [3,4,5,6,7]. In addition to the consequences of the hemorrhage on the outcome/mortality of patients with ICH, intensive care therapy addresses additional risk factors/comorbidities unrelated to the hemorrhage [8]. These may include prolonged mechanical ventilation and the need for renal replacement therapy, among other complications of intensive care [9,10,11].

Dehydration at the time of admission is often an important factor affecting expected short-term mortality in the assessment of other acute illnesses [12,13,14,15,16]. Thus, dehydration status can act as an imminent risk, particularly in elderly, secluded patients with an unclear duration of recumbency and/or eventual lengthy diagnostic procedures [17,18,19]. To date, there have been only sporadic reports on the influence of admission dehydration status on short-term mortality in patients with ICH [20].

We aimed to assess the potential influence of admission dehydration status on short-term mortality in patients with spontaneous, non-traumatic ICH.

## 2. Materials and Methods

From 2018 to 2019, all consecutive patients with spontaneous, non-traumatic ICH who were referred to our neurovascular center were recorded in a computerized database. Patients with ICH resulting from a potential underlying source of hemorrhage (e.g., arteriovenous malformation, aneurysm, tumor) were excluded from further analysis using a consistent diagnostic algorithm as previously reported [2]. After the identification of eligible patients, information was retrospectively obtained for each patient, comprising patient characteristics, pre-existing conditions, presence of anticoagulant/antiplatelet medication prior to ictus, localization and volume of ICH, neurological status on admission in terms of initial Glasgow Coma Scale (GCS), ICH score [3], as well as laboratory parameters at the time of admission. The ICH volume was determined using the abc/2 method of initial imaging [21]. Surgical treatment of patients studied consisted of cerebrospinal fluid diversion, ICH aspiration/evacuation, and/or decompressive craniectomy. The primary endpoint used was short-term mortality in terms of 30-day mortality.

Since no distinct definition of the dehydration status has been established, we adopted a definition including laboratory parameters and determined the dehydration status by the ratio of blood urea and creatinine [13,14,15,22]. In the present study, dehydration was defined as the prevalence of a urea-to-creatinine (U/Cr) ratio of >80 in the admission laboratory results.

## 3. Statistics

The computer software package SPSS (version 25, IBM Corp., Armonk, NY, USA) was used for data analysis. To compare continuous variables, the Mann–Whitney U test was selected when data were not normally distributed. An unpaired, two-sided t-test was used for parametric statistics after testing for normal distribution. Categorical variables were analyzed in contingency tables using Fisher’s exact test. Results with *p* < 0.05 were considered statistically significant.

To determine the independent predictors that might predict short-term mortality after experienced ICH and would be available at the time of admission, an additional multivariate analysis was performed using a binary logistic regression. A backward stepwise method was used to construct a multivariate logistic regression model with short-term mortality as the dependent variable, with an inclusion criterion for variables with presumed/proven clinical relevance.

## 4. Results

### 4.1. Patient Characteristics

A total of 249 patients with spontaneous ICH were identified using the above-mentioned inclusion criteria. The median age of all patients was 76 years (IQR 65–82 years), with 128 patients (51%) being female. Regarding the localization of ICH, supratentorial ICH appeared in 212 patients (85%) while infratentorial ICH was present in 37 patients (15%). At the time of admission, 127 patients (51%) exhibited a GCS ≤ 12, with 114 patients (46%) presenting with an intraventricular hemorrhage (IVH) component. With the combination of age and ICH volume, 201 patients (81%) demonstrated an ICH score ≤ 3 and 48 patients (19%) indicated an ICH score > 3. Furthermore, 41 patients (17%) presented with signs of cerebral herniation in terms of one or bilateral mydriasis as early as at the time of admission to our neurovascular center. A total of 62 patients with ICH (25%) displayed a midline shift of >5 mm during baseline radiological imaging. Overall, 92% of the chronologically trackable patients (156/169) received in-hospital imaging within the first h (≤1 h) following admission. In total, 32% of patients (*n* = 80) were referred from external hospitals with existing imaging and were therefore not included in the time data acquisition. A significant correlation between time from admission to first imaging and 30 days mortality was not detected (*p* = 0.2). As a result of spontaneous ICH, 65 patients (26%) underwent surgical intervention during the course of treatment (Table 1). However, surgical treatment was not associated with short-term mortality in patients with spontaneous ICH (39% of patients without surgical treatment versus 43% of patients with surgical treatment; *p* = 0.7). The median hospital length of stay was 8 days (IQR 4–18 days). Short-term mortality (30 days) was 41% (101 patients) in the overall patient cohort. Further details are provided in Table 1.

### 4.2. Admission Dehydration Status

**Figure 1:**
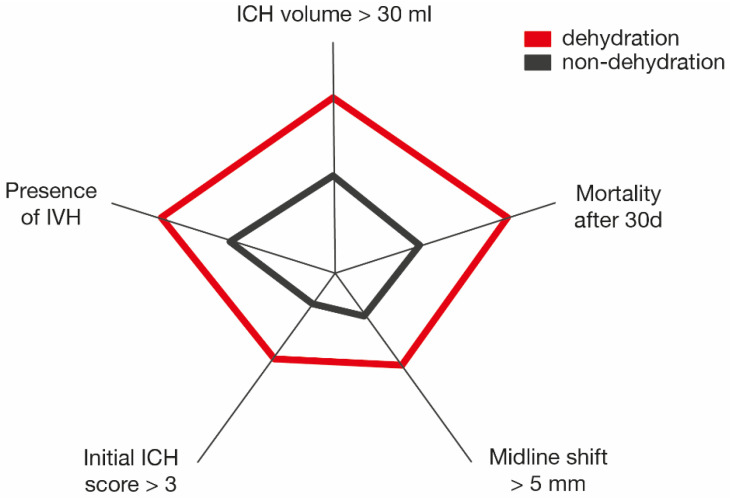
Graphical illustration of selected clinical and radiological conditions in dehydration/non-dehydration group and corresponding impact on mortality after 30 days. ICH, intracerebral hemorrhage; IVH, intraventricular hemorrhage.

Overall, 173 (69%) patients were assigned to the non-dehydrated subgroup and 76 (31%) to the dehydrated subgroup at the time of admission (Table 2). Diuretic drug treatment before ictus had no significant influence on the dehydration status of a patient with ICH (Table 1 and Table 2). The patients with ICH, who suffered from dehydration at the time of admission were significantly older compared with non-dehydrated patients with spontaneous ICH. Furthermore, patients with ICH and a dehydrated admission status were in a significantly deteriorated clinical condition (level of ICH score, presence of signs of decerebration, extent/volume of hemorrhage) compared to non-dehydrated patients with ICH (details are provided in Table 2, see also Figure 1).

### 4.3. Multivariate Analysis

An additional multivariate analysis identified “signs of cerebral herniation” (*p* = 0.008, OR 6.6, 95% CI 1.6–26.9), “initial midline shift > 5 mm” (*p* < 0.001, OR 8.2, 95% CI 3.4–19.6), “ICH score > 3” (*p* = 0.007, OR 3.9, 95% CI 1.4–10.5), and “admission dehydration status” (*p* = 0.007, OR 2.6, 95% CI 1.3–5.3) as independent predictors of short-term mortality in patients with spontaneous ICH (Nagelkerke’s R^2^ = 0.48).

## 5. Discussion

Dehydration represents an important component in the prevention of complications/mortality in a wide variety of diseases [15,23,24,25,26]. Dehydration, alongside fever, aspiration, and infection, is a potentially preventable complication of the course of several diseases and frequent in elderly patients [27,28]. Furthermore, initial dehydration at the time of admission seems to be a potential surrogate parameter for the severity in acute disorders and the subsequent clinical course. Especially in acute neurological disorders, which may be associated with reduced vigilance and thus reduced fluid intake, dehydration may have a negative influence on the further clinical course [29]. In this regard, an increased likelihood of thrombotic complications as well as the need for renal replacement therapy have been described repeatedly in patients suffering from ischemic stroke [25,30]. In patients with spontaneous ICH, the influence of dehydration status at the time of admission on the further clinical treatment course/outcome of the disease has not been identified conclusively.

The present study identified dehydration at the time of admission in 31% of patients admitted with spontaneous ICH to the authors’ neurovascular center. In this regard, the findings of the present study demonstrated that patients with admission dehydration status were of older age and presented with poorer neurological status. In addition, admission dehydration status was more frequently noted to occur in female patients with ICH than in male patients. As previously reported, this observation might arguably be explained by the lower muscle mass in women, which might lead to lower creatinine, but not urea, levels, thus negating the definition of dehydration utilized herein [31].

Factors associated with greater hematoma volume, such as a higher ICH score, signs of cerebral herniation, as well as increased MLS, were associated with an admission dehydration status in patients with ICH. While the underlying mechanism has not been clearly elucidated, Qureshi et al. suggested reduced cerebral perfusion due to hypovolemia in dehydrated patients with ICH as a reasonable explanation accounting for increasing perihematomal ischemia [32]. Contradictory theories advocate that hypovolemia-induced reduced blood pressure inhibits hematoma growth and dehydration-related hypernatremia increases intravascular osmolality causing a reduction in perihematomal edema and intracranial pressure [33,34,35,36]. Nevertheless, admission dehydration and poor outcome might be a simplified consequence of the fact that patients with lower GCS, greater ICH volume and therefore higher ICH score at admission might also be more neurologically impaired. However, neurologically impaired patients might be unable to adequately hydrate through the acute phase of deterioration, and in the case of increased age, occasionally suffer from an inadequate baseline hydration status already. In addition to dehydration, age might also act as a contributing factor to short-term mortality in patients with ICH, as elderly patients usually exhibit decreased renal function and are frailer compared to younger patients [37,38].

The assessment of a suspected dehydrated patient status using the laboratory values presented here is fast and performed with ease. However, a critique of such a definition, which relies solely on laboratory parameters, is that many additional aspects (especially in older, more frail patients) remain neglected. For example, other clinical parameters, urine analyses, or even ultrasound-based fluid volume analyses might provide a much more accurate impression of potential dehydration [39,40,41]. However, despite their accuracy, these methods might not be routinely applicable in the regular emergency patient. Nonetheless, the results of the current study might serve as a reason not only to define dehydration as such more precisely, but also to be able to better investigate suspected dehydration with established, more accurate methods after initial detection.

As mentioned previously, data regarding the association of admission dehydration status and short-term mortality in patients with spontaneous ICH remain scarce. To date, only one registry analysis has suggested an association between both parameters [20]. Gao et al. found that dehydration reduces in-hospital mortality in patients with ICH, indicating an inverse effect of admission dehydration status compared with the present cohort analysis [20]. However, as it was a registry analysis, details of ICH (e.g., location, cause, volume) were not accessible in the previous study [20]. Thus, other causes of ICH such as aneurysms, arteriovenous malformations, trauma, and tumors were not excluded, and the generalizability of the results of this previously published work remained limited. In addition, Gao et al. stated, in their limitation section, that neither imaging data nor information on the severity of ICH on admission was available during the registry analysis [20]. Through a meticulous consideration of patients, the results of the present study suggest that admission dehydration status in patients with spontaneous ICH may have a direct or indirect effect on the clinical course and short-term mortality, similarly to other diseases. In line with this reasoning, the multivariate regression analysis of the present study identified admission dehydration status in patients with spontaneous ICH as an independent predictor of short-term mortality.

## 6. Conclusions

The results of the present study suggest that admission dehydration status might serve as a significant and independent predictor of short-term mortality in patients of spontaneous, non-traumatic ICH.

## 7. Limitations

Interpretation of the results of the present study must be made in consideration of several shortcomings. In addition to the retrospective data collection method, the present study solely considers clinical factors that were known at admission for an interpretation of results. This may result in the neglection of potential outcome determinants in the further course of treatment (e.g., postoperative and/or intensive care complications). However, the retrospective aspect of the present study also restricts the informative value of the data on admission parameters. For instance, missing information in medical records limits the validity of data for the exact time course of further diagnostic measures/treatment (e.g., time from admission/ictus to first imaging). Furthermore, the inconsistent definition of dehydration throughout the literature is a significant limitation. The present study used the widely accepted U/Cr-ratio, but this may vary in patients with other medical conditions that were not further assessed in the present study. Nevertheless, this detailed exploration of a selected, but nonetheless consecutive, patient population demonstrates, for the first time, the potentially negative impact of dehydration at admission on outcome in patients with ICH. For a broader interpretation and possible clinical implications, additional studies are needed to supplement this study and improve the dataset in this context.

## Figures and Tables

**Table 1:** Overall patient characteristics. ICH, intracerebral hemorrhage; IQR, interquartile range; GCS, Glasgow Coma Scale; IVH, intraventricular hemorrhage; MLS, midline shift.

	Patients with Spontaneous ICH, *n* = 249
Median age (years, IQR)	76 (65–82)
Female sex	128 (51%)
Anticoagulation/antiplatelet medication prior ictus	125 (50%)
Pre-existing hypertension	204 (82%)
Pre-existing diuretic treatment	77 (31%)
Initial ICH score > 3	48 (19%)
GCS ≥ 13	122 (49%)
patient age ≥ 80 years	83 (33%)
infratentorial location	37 (15%)
ICH volume ≥ 30 mL	105 (42%)
presence of IVH	114 (46%)
Presence of clinical signs of herniation at admission	41 (17%)
MLS > 5 mm	62 (25%)
Surgical treatment	65 (26%)
Short-term mortality	101 (41%)

**Table 2:** Patient characteristics in non-dehydrated and dehydrated group with ICH.

	Non-Dehydration U/Cr ≤ 80, *n* = 173	Dehydration U/Cr > 80, *n* = 76	
Median age (years, IQR)	75 (63–82)	76 (70–82)	*p* < 0.001
Female sex	77 (45%)	51 (67%)	*p* = 0.001, OR
Anticoagulation/antiplatelet medication prior ictus	86 (50%)	39 (51%)	*p* = 0.9
Pre-existing hypertension	140 (81%)	64 (84%)	*p* = 0.6
Pre-existing diuretic treatment	56 (32%)	21 (28%)	*p* = 0.6
Supratentorial ICH location	151 (87%)	61 (80%)	*p* = 0.2
ICH volume ≥ 30 mL	59 (34%)	46 (61%)	*p* < 0.001, OR 3.0, 95% CI 1.7–5.2
Presence of IVH	66 (38%)	48 (63%)	*p* < 0.001, OR 2.8, 95% CI 1.6–4.9
Initial ICH score > 3	21 (12%)	27 (36%)	*p* < 0.001, OR 3.9, 95% CI 2.1–7.7
Presence of clinical signs of herniation at admission	21 (12%)	20 (26%)	*p* = 0.009, OR 2.6, 95% CI 1.3–5.1
MLS > 5 mm	32 (18%)	30 (39%)	*p* = 0.001, OR 2.9, 95% CI 1.6–5.2
Short-term mortality	53 (31%)	48 (63%)	*p* < 0.001, OR 3.9, 95% CI 2.2–6.8

## Data Availability

The original contributions presented in the study are included in the article, further inquiries can be directed to the corresponding author.

## References

[1] Hemphill J.C., Greenberg S.M., Anderson C.S., Becker K., Bendok B.R., Cushman M., Fung G.L., Goldstein J.N., Macdonald R.L., Mitchell P.H. (2015). Guidelines for the Management of Spontaneous Intracerebral Hemorrhage: A Guideline for Healthcare Professionals from the American Heart Association/American Stroke Association. Stroke.

[2] Schuss P., Bode C., Borger V., Coch C., Guresir A., Hadjiathanasiou A., Hamed M., Kuchelmeister K., Lehmann F., Muller M. (2021). MR-Imaging and Histopathological Diagnostic Work-Up of Patients with Spontaneous Lobar Intracerebral Hemorrhage: Results of an Institutional Prospective Registry Study. Diagnostics.

[3] Hemphill J.C., Bonovich D.C., Besmertis L., Manley G.T., Johnston S.C. (2001). The ICH score: A simple, reliable grading scale for intracerebral hemorrhage. Stroke.

[4] Fung C., Murek M., Z’Graggen W.J., Krahenbuhl A.K., Gautschi O.P., Schucht P., Gralla J., Schaller K., Arnold M., Fischer U. (2012). Decompressive hemicraniectomy in patients with supratentorial intracerebral hemorrhage. Stroke.

[5] Hadjiathanasiou A., Schuss P., Ilic I., Borger V., Vatter H., Guresir E. (2018). Decompressive craniectomy for intracerebral haematoma: The influence of additional haematoma evacuation. Neurosurg. Rev..

[6] Divani A.A., Liu X., Di Napoli M., Lattanzi S., Ziai W., James M.L., Jafarli A., Jafari M., Saver J.L., Hemphill J.C. (2019). Blood Pressure Variability Predicts Poor In-Hospital Outcome in Spontaneous Intracerebral Hemorrhage. Stroke.

[7] Gessler F., Schmitz A.K., Dubinski D., Bernstock J.D., Lehmann F., Won S.Y., Wittstock M., Guresir E., Hadjiathanasiou A., Zimmermann J. (2021). Neurosurgical Considerations Regarding Decompressive Craniectomy for Intracerebral Hemorrhage after SARS-CoV-2-Vaccination in Vaccine Induced Thrombotic Thrombocytopenia-VITT. J. Clin. Med..

[8] Faigle R., Chen B.J., Krieger R., Marsh E.B., Alkhachroum A., Xiong W., Urrutia V.C., Gottesman R.F. (2021). Novel Score for Stratifying Risk of Critical Care Needs in Patients with Intracerebral Hemorrhage. Neurology.

[9] Lehmann F., Schenk L.M., Ilic I., Putensen C., Hadjiathanasiou A., Borger V., Zimmermann J., Guresir E., Vatter H., Bode C. (2021). Prolonged Mechanical Ventilation in Patients with Deep-Seated Intracerebral Hemorrhage: Risk Factors and Clinical Implications. J. Clin. Med..

[10] Schenk L.M., Schneider M., Bode C., Guresir E., Junghanns C., Müller M., Putensen C., Vatter H., Zimmermann J., Schuss P. (2021). Early Laboratory Predictors for Necessity of Renal Replacement Therapy in Patients With Spontaneous Deep-Seated Intracerebral Hemorrhage. Front. Neurol..

[11] Peng J., Volbers B., Sprugel M.I., Hoelter P., Engelhorn T., Jiang Y., Kuramatsu J.B., Huttner H.B., Dorfler A., Schwab S. (2021). Influence of Early Enteral Nutrition on Clinical Outcomes in Neurocritical Care Patients With Intracerebral Hemorrhage. Front. Neurol..

[12] Liu K., Pei L., Gao Y., Zhao L., Fang H., Bunda B., Fisher L., Wang Y., Li S., Li Y. (2019). Dehydration Status Predicts Short-Term and Long-Term Outcomes in Patients with Cerebral Venous Thrombosis. Neurocrit. Care.

[13] Lacey J., Corbett J., Forni L., Hooper L., Hughes F., Minto G., Moss C., Price S., Whyte G., Woodcock T. (2019). A multidisciplinary consensus on dehydration: Definitions, diagnostic methods and clinical implications. Ann. Med..

[14] Rowat A., Graham C., Dennis M. (2012). Dehydration in hospital-admitted stroke patients: Detection, frequency, and association. Stroke.

[15] Liu C.H., Lin S.C., Lin J.R., Yang J.T., Chang Y.J., Chang C.H., Chang T.Y., Huang K.L., Ryu S.J., Lee T.H. (2014). Dehydration is an independent predictor of discharge outcome and admission cost in acute ischaemic stroke. Eur. J. Neurol..

[16] Schrock J.W., Glasenapp M., Drogell K. (2012). Elevated blood urea nitrogen/creatinine ratio is associated with poor outcome in patients with ischemic stroke. Clin. Neurol. Neurosurg..

[17] Miller H.J. (2015). Dehydration in the Older Adult. J. Gerontol. Nurs..

[18] Palevsky P.M., Bhagrath R., Greenberg A. (1996). Hypernatremia in hospitalized patients. Ann. Intern. Med..

[19] Warren J.L., Bacon W.E., Harris T., McBean A.M., Foley D.J., Phillips C. (1994). The burden and outcomes associated with dehydration among US elderly, 1991. Am. J. Public Health.

[20] Gao B., Gu H., Yu W., Liu S., Zhou Q., Kang K., Zhang J., Li Z., Zhao X., Wang Y. (2021). Admission Dehydration is Associated with Significantly Lower In-Hospital Mortality after Intracerebral Hemorrhage. Front. Neurol..

[21] Kothari R.U., Brott T., Broderick J.P., Barsan W.G., Sauerbeck L.R., Zuccarello M., Khoury J. (1996). The ABCs of measuring intracerebral hemorrhage volumes. Stroke.

[22] Kelly J., Hunt B.J., Lewis R.R., Swaminathan R., Moody A., Seed P.T., Rudd A. (2004). Dehydration and venous thromboembolism after acute stroke. QJM.

[23] Aasbrenn M., Christiansen C.F., Esen B.O., Suetta C., Nielsen F.E. (2021). Mortality of older acutely admitted medical patients after early discharge from emergency departments: A nationwide cohort study. BMC Geriatr..

[24] Cortes-Vicente E., Guisado-Alonso D., Delgado-Mederos R., Camps-Renom P., Prats-Sanchez L., Martinez-Domeno A., Marti-Fabregas J. (2020). Corrigendum: Frequency, Risk Factors, and Prognosis of Dehydration in Acute Stroke. Front. Neurol..

[25] Elias S., Hoffman R., Saharov G., Brenner B., Nadir Y. (2016). Dehydration as a Possible Cause of Monthly Variation in the Incidence of Venous Thromboembolism. Clin. Appl. Thromb./Hemost..

[26] Saadatnia M., Fatehi F., Basiri K., Mousavi S.A., Mehr G.K. (2009). Cerebral venous sinus thrombosis risk factors. Int. J. Stroke.

[27] Paulis S.J.C., Everink I.H.J., Halfens R.J.G., Lohrmann C., Schols J. (2018). Prevalence and Risk Factors of Dehydration among Nursing Home Residents: A Systematic Review. J. Am. Med. Dir. Assoc..

[28] Bunn D., Jimoh F., Wilsher S.H., Hooper L. (2015). Increasing fluid intake and reducing dehydration risk in older people living in long-term care: A systematic review. J. Am. Med. Dir. Assoc..

[29] Buoite Stella A., Gaio M., Furlanis G., Ridolfi M., Ajcevic M., Sartori A., Caruso P., Morrison S.A., Naccarato M., Manganotti P. (2020). Prevalence of hypohydration and its association with stroke severity and independence outcomes in acute ischemic stroke patients. J. Clin. Neurosci..

[30] Kim H., Lee K., Choi H.A., Samuel S., Park J.H., Jo K.W. (2017). Elevated Blood Urea Nitrogen/Creatinine Ratio Is Associated with Venous Thromboembolism in Patients with Acute Ischemic Stroke. J. Korean Neurosurg. Soc..

[31] McPherson K., Healy M.J., Flynn F.V., Piper K.A., Garcia-Webb P. (1978). The effect of age, sex and other factors on blood chemistry in health. Clin. Chim. Acta.

[32] Qureshi A.I. (2008). Acute hypertensive response in patients with stroke: Pathophysiology and management. Circulation.

[33] Frey M.A., Lathers C., Davis J., Fortney S., Charles J.B. (1994). Cardiovascular responses to standing: Effect of hydration. J. Clin. Pharmacol..

[34] Diringer M.N., Scalfani M.T., Zazulia A.R., Videen T.O., Dhar R. (2011). Cerebral hemodynamic and metabolic effects of equi-osmolar doses mannitol and 23.4% saline in patients with edema following large ischemic stroke. Neurocrit. Care.

[35] Qureshi A.I., Wilson D.A., Traystman R.J. (1999). Treatment of elevated intracranial pressure in experimental intracerebral hemorrhage: Comparison between mannitol and hypertonic saline. Neurosurgery.

[36] Qureshi A.I., Wilson D.A., Traystman R.J. (2002). Treatment of transtentorial herniation unresponsive to hyperventilation using hypertonic saline in dogs: Effect on cerebral blood flow and metabolism. J. Neurosurg. Anesthesiol..

[37] Fried L.P., Tangen C.M., Walston J., Newman A.B., Hirsch C., Gottdiener J., Seeman T., Tracy R., Kop W.J., Burke G. (2001). Frailty in older adults: Evidence for a phenotype. J. Gerontol. A Biol. Sci. Med. Sci..

[38] Radholm K., Arima H., Lindley R.I., Wang J., Tzourio C., Robinson T., Heeley E., Anderson C.S., Chalmers J., Investigators I. (2015). Older age is a strong predictor for poor outcome in intracerebral haemorrhage: The INTERACT2 study. Age Ageing.

[39] Fortes M.B., Owen J.A., Raymond-Barker P., Bishop C., Elghenzai S., Oliver S.J., Walsh N.P. (2015). Is this elderly patient dehydrated? Diagnostic accuracy of hydration assessment using physical signs, urine, and saliva markers. J. Am. Med. Dir. Assoc..

[40] Shokoohi H., Berry G.W., Shahkolahi M., King J., King J., Salimian M., Poshtmashad A., Pourmand A. (2017). The diagnostic utility of sonographic carotid flow time in determining volume responsiveness. J. Crit. Care.

[41] Bahouth M.N., Gottesman R.F., Szanton S.L. (2018). Primary ‘dehydration’ and acute stroke: A systematic research review. J. Neurol..

